# Supplementary material for: Highly efficient three-dimensional solar evaporator for high salinity desalination by localized crystallization
Source: Nat Commun. 2020 Jan 27;11:521. doi: 10.1038/s41467-020-14366-1 (PMC6985111; doi:10.1038/s41467-020-14366-1)
Supplement: Supplementary file 1 — Supplementary Information [file 41467_2020_14366_MOESM1_ESM.pdf]

Supplementary Information

Highly efficient 3D solar evaporator for high salinity desalination by localized crystallization

Wu et al.

Supplementary Figure 1

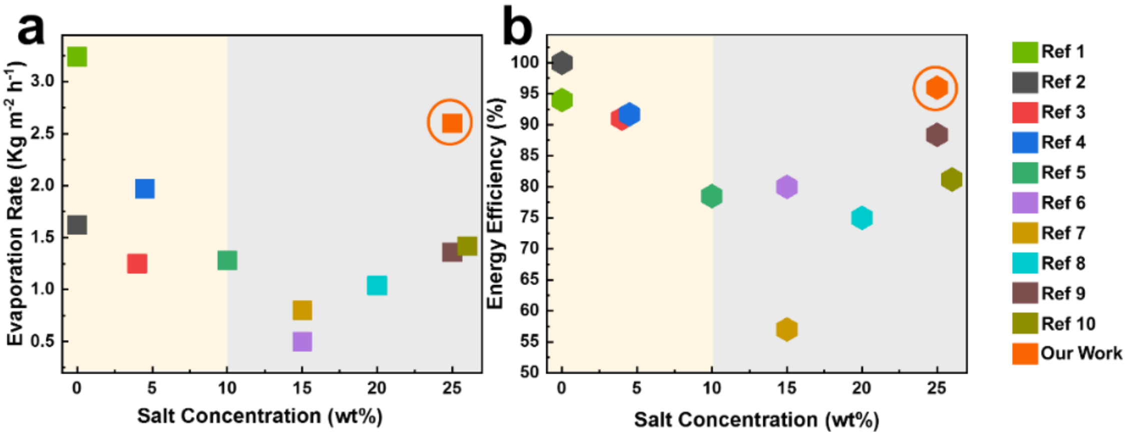

Supplementatry Figure 1 | Comparison of solar desalination performance and previous reports under one sun illumination, including the evaporation rate (a) and energy efficiency (b) with the variation of salt concentration.

Supplementary Figure 2

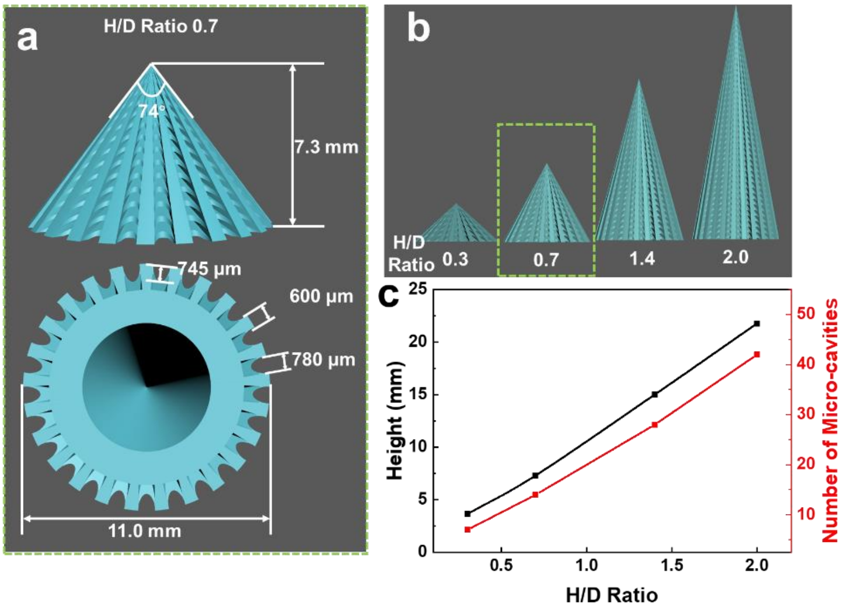

Supplementatry Figure 2 | Scheme of the detailed dimension of the designed bio-mimetic 3D evaporator. **a**. Scheme of the bio-mimetic 3D model with a height-to-diameter (H/D) ratio of 0.7. **b**. Scheme of the bio-mimetic 3D models with different H/D ratios. **c**. Bio-mimetic 3D evaporator height and corresponding numbers of micro-cavities along each groove of the 3D structures with different H/D ratios. In detail, four 3D structures with different H/D ratios were designed, which includes the 3D evaporators with the same initial micro-cavity dimension but different structure heights and different numbers of micro-cavities along each groove. (H/D ratio of 0.3, 0.7, 1.4 and 2.0 represent the structure with a height of 3.6 mm, 7.3 mm, 15.0 mm and 21.8 mm, and with numbers of micro-cavities along each groove of 7, 14, 28 and 42, respectively).

Supplementary Figure 3

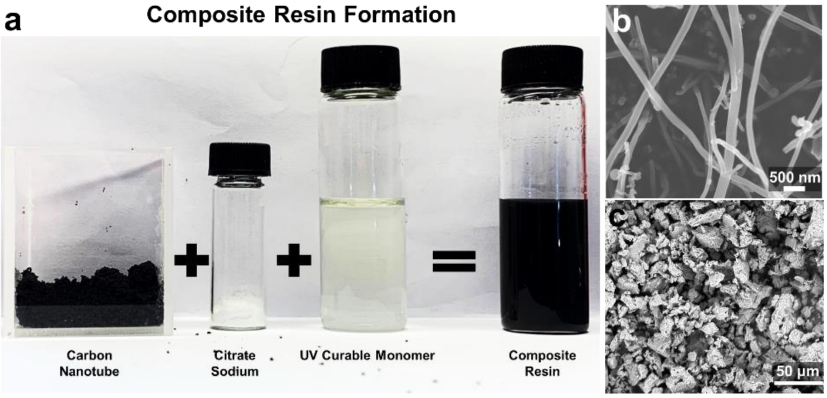

Supplementatry Figure 3 | **a**. The formation of the composite resin for size-dependent resin refilling induced 3D printing system. **b.** SEM characterization of CNTs. **c.** SEM characterization of citrate sodium particles.

Supplementary Figure 4

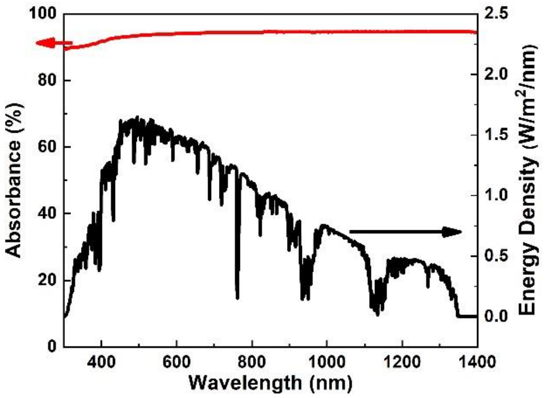

Supplementatry Figure 4 | UV-vis spectra of the composite plane film prepared from the composite resin.

Supplementary Figure 5

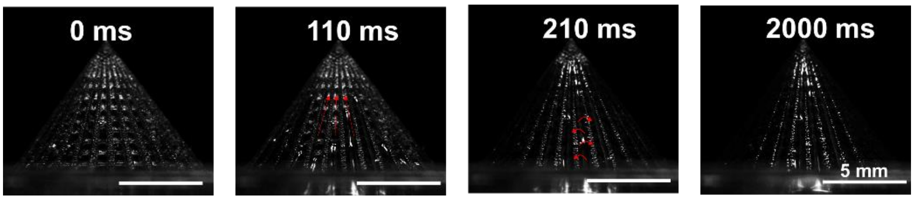

Supplementatry Figure 5 | Time sequence of optical captures of the two-step water precursor directionally upward moving process on the 3D structure without surface distributed micropores.

Supplementary Figure 6

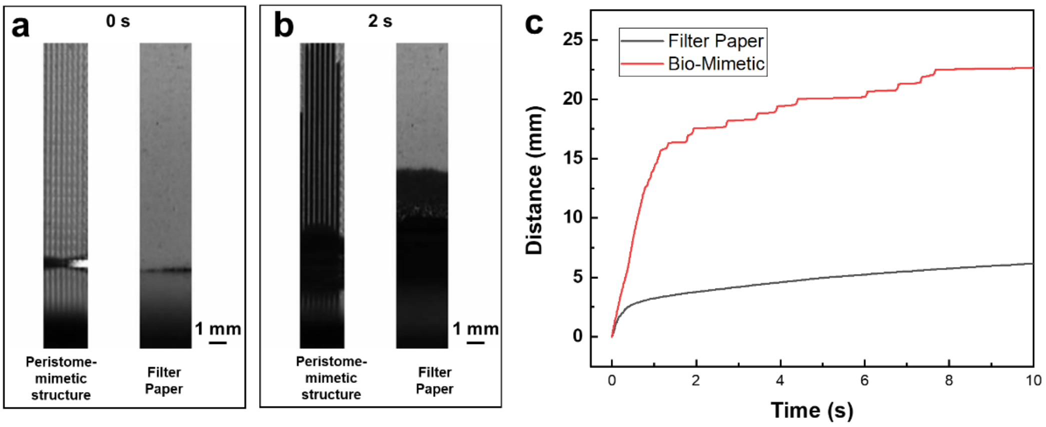

Supplementatry Figure 6 | The water upward moving process on the bio-mimetic structure and the filter paper. **a** and **b** are optical images of the structures before contacting with water and after contacting with water for 2 seconds, respectively. **c**. The distance of the water precursor moving on the bio-mimetic structure and filter paper along with time. Black and red lines represent the water precursor moving distance on the filter paper and the bio-mimetic structure, respectively. The water transport inside the filter paper is based on the porous structures induced capillary wicking, whose speed is inhibited by the small porous structures of the filter paper. However, water transportation on our bio-mimetic structure is a kind of surface water transport that based on the continuous filling of the micro-cavities array along the sidewall. The micro-cavity, which is the standard structure of the peristome surface of the pitcher plant, allows continuous and inward liquid transport. Liquid spreads along the micro-cavity with an accelerating speed as the precursor approaching the apex of the cavity and overflow into the next micro-cavity to repeat the spreading process, leading to a step-like transport behavior. Comparing with the water inside filter paper, water shows an ultra-fast water transport speed on the biomimetic structure.

Supplementary Figure 7

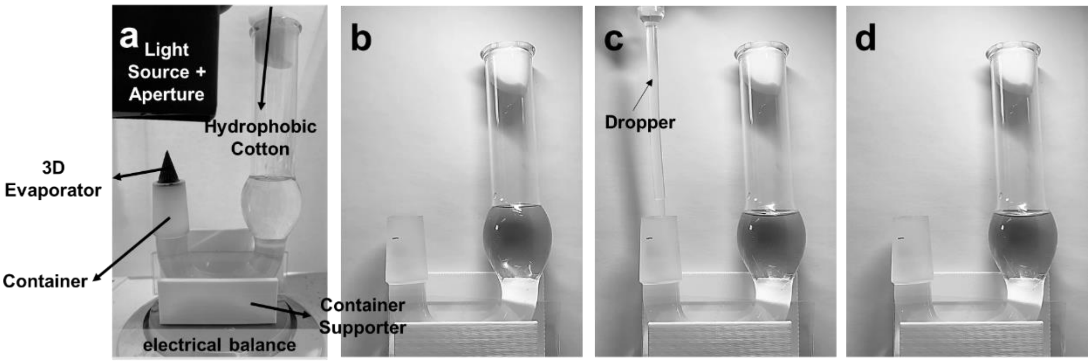

Supplementatry Figure 7 | **a**. Optical image of the experimental apparatus for measuring the evaporation rate in the open system. **b** - **d**. Optical captures of the process of extracting liquid from the left side of the U-shaped tube. The liquid height of the left side remains almost unchanged after extracting liquid from the left side. The container used is a U-shaped glass tube for the open system. The left side of the U-shaped glass tube is straight (left side, Supplementary Figure 7a), while the opening of the right side is higher than the left side. Besides, the right side possesses a spherical protrusion (right side, Supplementary Figure 7a) at the same height with the opening of the left side. The diameter of the spherical protrusion is about 2.5 times the diameter of the straight left side. For an open system, the heights of the liquid surfaces on both sides should be the same ascribing from the property of the U-shaped tube. Therefore, extracting liquid from one side, the liquid will be supplemented from the other side until reaching the same liquid surface height on both sides (Supplementary Figure 7b - 7d). Calculating from the solar steam generation rate of the bio-mimetic 3D evaporator, the liquid height can be kept almost unchanged for at least 4 hours ascribing from the large spherical protrusion on the right side. The self-float 3D evaporator will not fall inside the tube during the measuring process. Therefore, the effective solar steam generation surface of the bio-mimetic 3D evaporator in the open system is considered to be in free contact with the surrounding air and is not sheltered or influenced by the sidewall of the container. In addition, the opening of the right

side of the U-shaped tube is filled with hydrophobic-treated cotton to inhibit water evaporation from the right side.

Supplementary Figure 8

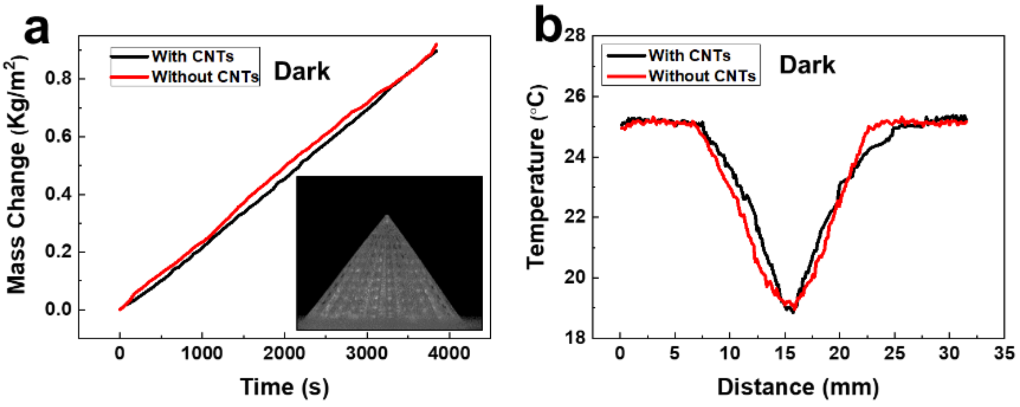

Supplementatry Figure 8 | **a**. Mass change of the water on the bio-mimetic 3D evaporator and the bio-mimetic 3D structure prepared from the composite resin without the addition of CNTs in darkness. Inset is the optical image of the 3D structure prepared from composite resin without the addition of CNTs. **b**. Temperature profiles along the sidewall of the 3D evaporators with and without the addition of CNTs in darkness.

Supplementary Figure 9

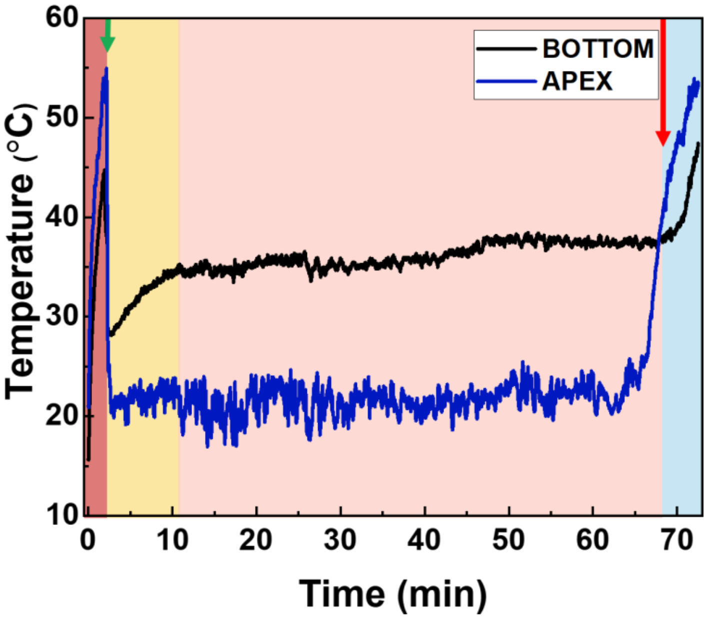

Supplementatry Figure 9 | Temperature evolution along with the time of the apex and bottom position on the bio-mimetic 3D evaporator. The green arrow indicates the introduction of water on the 3D evaporator. Red arrow demonstrated the exhaustion of water on the 3D evaporator.

Supplementary Figure 10

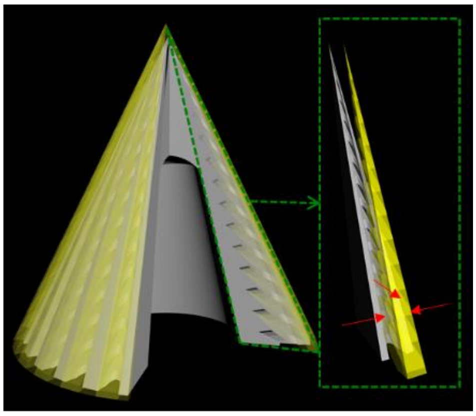

Supplementatry Figure 10 | Scheme of the contact mode of each groove of water with the corresponding groove on the bio-mimetic 3D evaporator. Each groove of water is surrounded by the three connected sidewalls of the corresponding groove, where annular heating from three directions of the groove structure occurs (red arrows). The designed groove structure and the contacting mode of grooves of water with corresponding grooves contributed to effective energy transfer from the evaporator to the liquid film.

Supplementary Figure 11

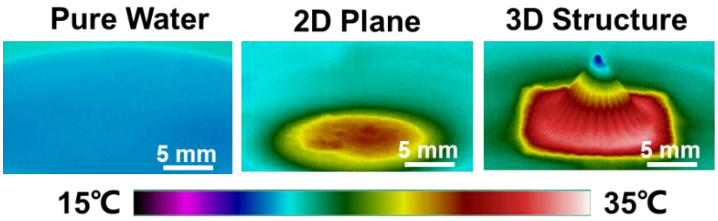

Supplementatry Figure 11 | Infrared images of the dynamic equilibrium states of pure water, the surface of the 2D plane and the bio-mimetic 3D evaporator under one sun illumination.

Supplementary Figure 12

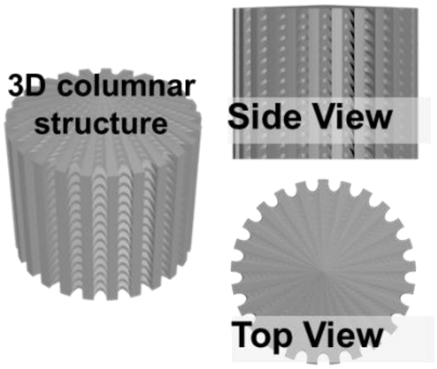

Supplementatry Figure 12 | Scheme of the detailed morphology of the 3D columnar structure. The 3D columnar structure possesses 25 grooves of micro-cavity arrays along the sidewall of the columnar structure. The two structures possess the same projected area used for calculating the water evaporation rate and the same height (the same H/D ratio). The sidewall and upper surface of the 3D columnar structure are comprised of 25 grooves of micro-cavity arrays without asymmetry. Due to the columnar structure, micro-cavities along each groove of the sidewall possesses the same dimension without gradient. The top surface of the 3D columnar structure is designed with 25 radially patterned asymmetric grooves composed of micro-cavity arrays with the same height as the micro-cavity on the sidewall for sustaining the continuous liquid film. Otherwise, water will dewet on the top surface during water evaporation, which will decrease the efficient water/structure contact area. The liquid film generated on the 3D columnar structure is homogeneous both on the sidewall and the top surface without the thickness gradient. The 3D columnar structure is also prepared from the size-dependent resin refilling induced 3D printing through employing the composite resin in Supplementary Figure 3 with the same post 3D printing treatment procedure.

Supplementary Figure 13

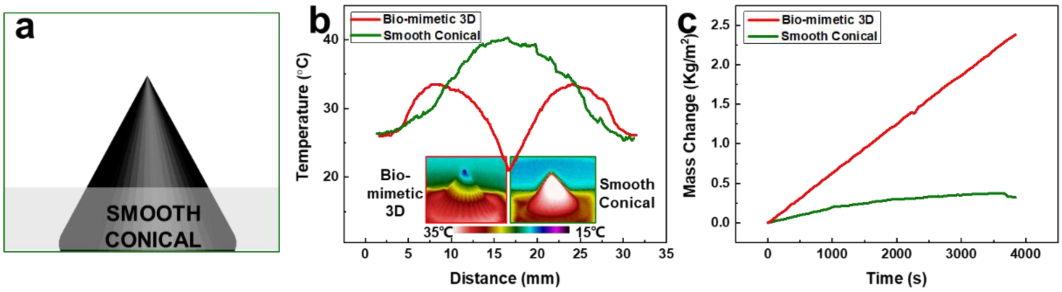

Supplementatry Figure 13 | **a**. Scheme of the smooth conical structure. The structure possesses the same dimension with the bio-mimetic 3D structure with the only difference in the sidewall morphology. **b**. Temperature profiles of the bio-mimetic 3D evaporator (red line) and the smooth conical structure (green line) under one sun illumination. Insets are corresponding infrared images showing the temperature distribution at the equilibrium state on the bio-mimetic 3D structure and the smooth conical structure under one sun illumination. **c**. The mass change of the water on the bio-mimetic 3D structure (red line) and the smooth conical structure (green line) under one sun illumination. The smooth conical structure is prepared from the size-dependent resin refilling induced 3D printing through employing the composite resin in Supplementary Figure 3.

Supplementary Figure 14

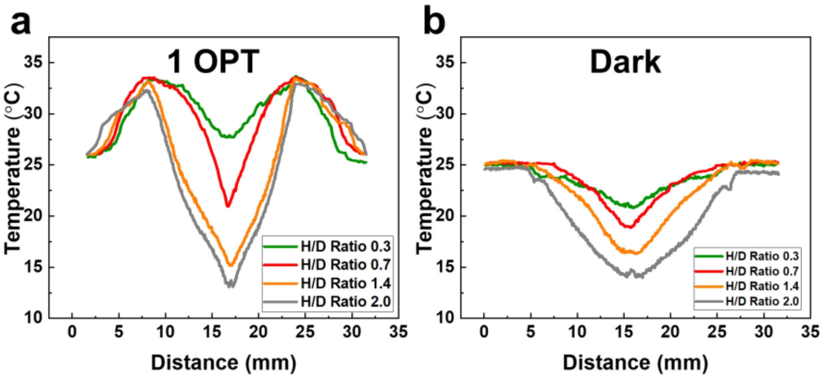

Supplementatry Figure 14 | **a**. Temperature profiles along the bio-mimetic 3D evaporators with different H/D ratios under one sun illumination. **b**. Temperature profiles along the bio-mimetic 3D evaporators with different H/D ratios in darkness. Green, red, yellow and grey lines represent the bio-mimetic evaporators with height-to-diameter ratios of 0.3, 0.7, 1.4 and 2.0, respectively.

Supplementary Figure 15

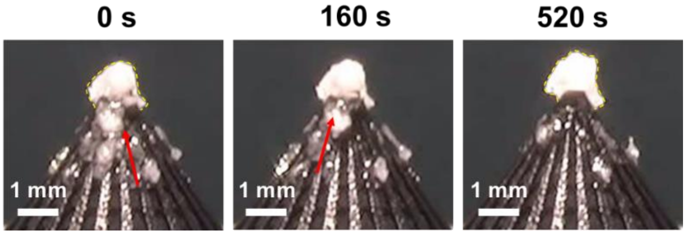

Supplementatry Figure 15 | Optical captures of the salt crystallized on the sidewall of the bio-mimetic 3D evaporator moving along with the supplemented water film upward to the apex position.

Supplementary Figure 16

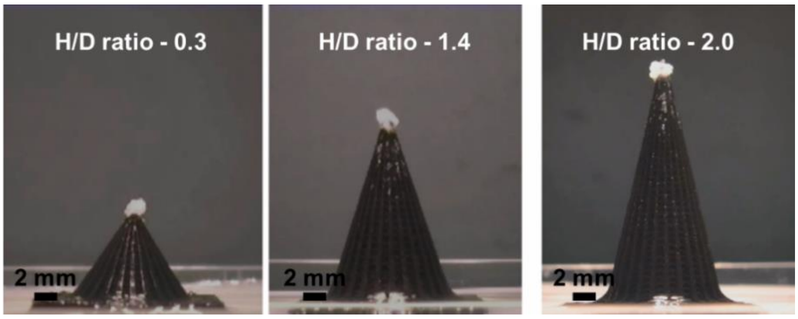

Supplementatry Figure 16 | Optical captures of the localized crystallization phenomenon on bio-mimetic 3D evaporators with different H/D ratios under one sun illumination.

Supplementary Figure 17

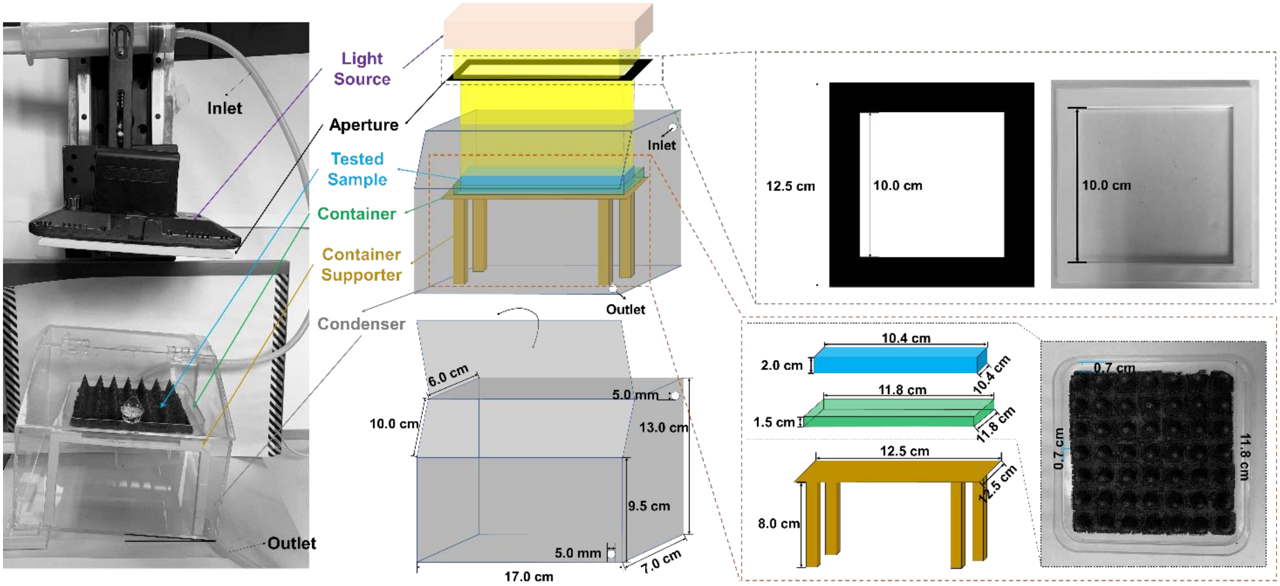

Supplementatry Figure 17 | Optical images and corresponding geometric parameters of the closed system used for measuring the water collection rate. The container is a polyethylene coverless box with a dimension of 11.8 cm × 11.8 cm, while the dimension of the printed 3D evaporator array is 10.4 cm × 10.4 cm, whose intervals are designed to fill with flat surfaces. The supplementation of brine water is through the inlet, and the water collecting rate is calculated through the amount of water collected from the outlet per three hours. The closed system is a much more complex system compared with the open system, many parasitics, e.g., the increased humidity inside the condenser, the condensation of vapor on the top and sidewall of the condenser, the fall down of the condensed water from the top and sidewall of the condenser, and the supplementation velocity of source water from the inlet, *etc.,* may influence the water evaporation rate. Therefore, for the closed system, we measured the water collecting rate rather than the water evaporation rate.

Supplementary Figure 18

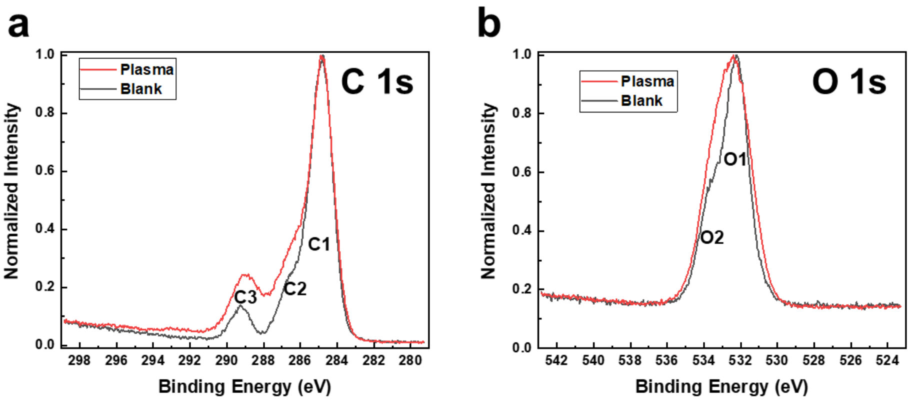

Supplementatry Figure 18 | **a**. XPS spectra of C 1s peaks of the upper surface of the bio-mimetic 3D evaporator before (black line) and after (red line) plasma treatment. C1 at a binding energy of 284.8 eV corresponds to the C*–C* and C*–H bond. C2 at 286.4 eV reflects the C*–O bond, and C3 at 288.9 eV corresponds to O=C*–O bond. **b**. XPS spectra of O 1s peaks of the upper surface of the bio-mimetic 3D evaporator before (black line) and after (red line) plasma treatment. O1 at a binding energy of 532.2 eV corresponds to C=O* bond and O2 at 533.6 eV represents O*–C=O bond. After plasma treatment, the atomic ratio of oxygen increases from 21.7% to 33.11% after plasma treatment, which means that the number of oxygen-containing groups rises. In addition, the C 1s and O 1s spectra showed increase in the surface concentration of O–C=O functional groups as well as enrichment of C–O groups on the structure's surface after plasma treatment, which contributes fundamentally to the improvement of surface hydrophilicity[17].

Supplementary Figure 19

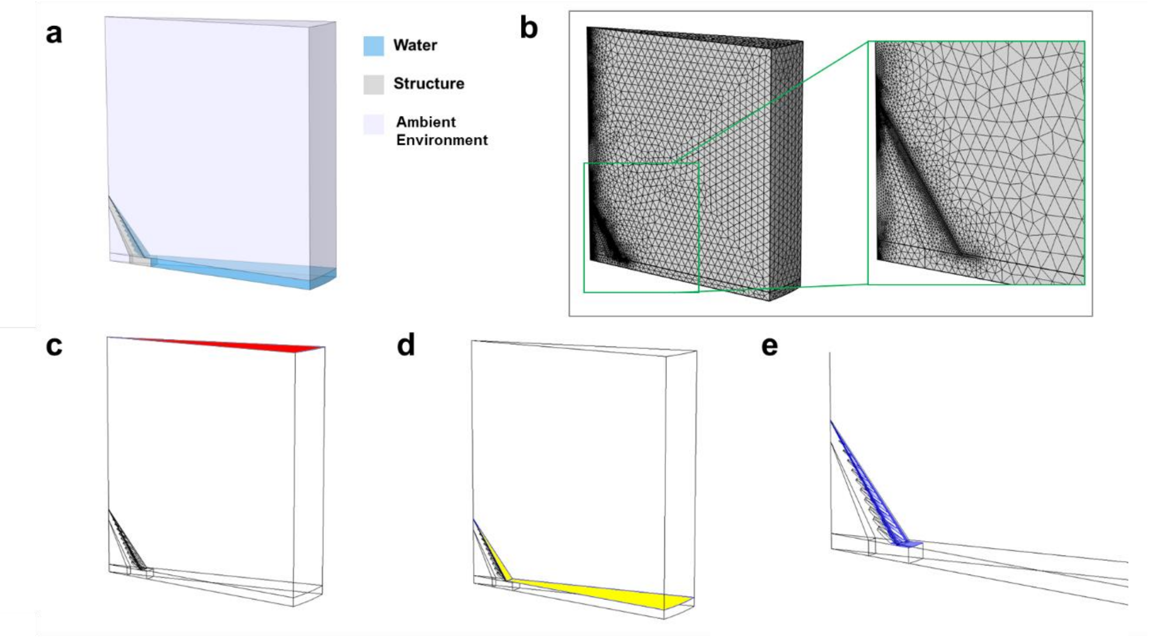

Supplementatry Figure 19 | Detailed geometrical conditions of the simulation. **a**. Scheme of the model used for numerical simulation of the wet bio-mimetic 3D structure in darkness and under one sun illumination. Because of the symmetry of the structure, one groove of the wet bio-mimetic structure in darkness and under one sun illumination is simulated. **b**. Mesh conditions of the simulation model. The maximum mesh size is 1 mm and the minimum of the mesh size is 0.05 mm. Mesh is refined at the boundaries of water film surface with the highest resolution. **c**. The red boundary represents the boundary of the ambient environment and is set as 293.15 K and relative humidity 0.5. **d**. The yellow boundary represents the water film surface and is set as relative humidity 0.5 and latent heat source by evaporation. **e**. The blue boundary which represents the boundary of the interface of the 3D structure and the water film. Under one sun illumination, it exits as the heat source of 0.5 W m^{-2}, while in darkness, it exists as not energy output. Other undefined boundaries are set as zero flux of energy or steam.

Supplementary Figure 20

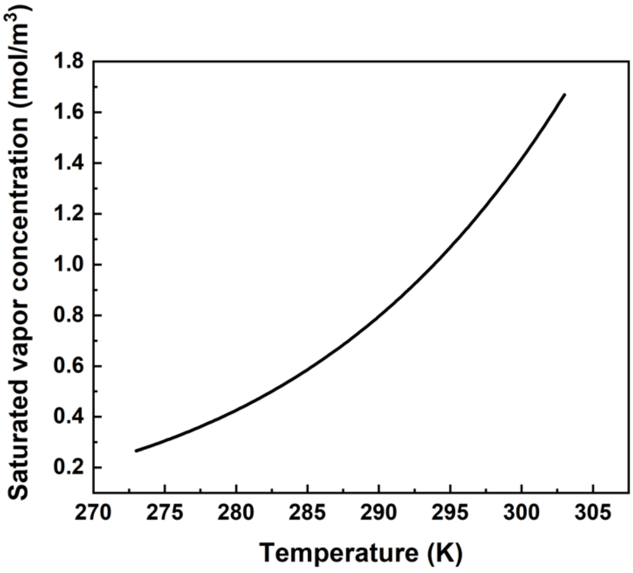

Supplementatry Figure 20 | The plot of saturated vapor concentration with the variation of temperature.

Supplementary Table 1

Supplementary Table 1 | Summary of representative references on solar-driven desalination with the variation of salt concentration [1-16].

Ref.	Salt Concentration (wt%)	Mass Change (kg m^{-2} h^{-1})	Energy efficiency (%)	Salt removing capability	Salt rejecting property	Notes
1	0	3.2	~ 94	/	/	Reduced water vaporization enthalpy, with evaporation rate of 3.2 kg m^{-2} h^{-1}, no data on high-salinity samples
2	0	1.62	100	/	/	/
3	~ 3.5	1.25	91	/	/	Commercial synthetic seawater
4	~ 4.5	1.97	91.7	/	/	seawater sample from Nanhai Sea
5	10	1.28	78.5	/	√	Brine sample: NaCl solution
6	15	0.8	57	/	√	Brine sample: NaCl solution
7	15	0.5	~ 80	/	/	Brine sample: NaCl solution
8	20	1.04	75.0	/	√	Brine sample: NaCl solution
9	25	1.36	88.4	/	/	Brine sample: NaCl solution
10	26	1.42	81.2	√	/	Brine sample: NaCl solution; Localized crystallization, salt falls down due to gravity
11	0	1.3	72	/	/	20wt% NaCl solution capable, without data
12	0	2.12	91.5	/	/	The data are measured under reduced pressure (0.25 atm)
13	1.19	1.99	/	/	/	Energy efficiency for pure water is ~100%, 1.19 wt% is the solid content inside the wastewater

14	3.5	2.5 kg m^{-2} day^{-1}	56 ± 2.5	/	√	Brine sample: NaCl solution, with NaCl solid on the evaporator
15	~ 10	2.5	~ 95	/	√	Reduced water vaporization enthalpy, Dead Sea water sample
16	21	1.94	89.9	/	/	Brine sample: NaCl solution
Our work	25	2.24 (H/D 0.7)<br>2.6 (H/D 1.4)	> 96	√	/	Brine sample: NaCl solution; Localized crystallization, free standing salt

Supplementaru Table 2

Supplementary Table 2 | Comparison of the solar-driven water evaporation parameters of the bio-mimetic 3D evaporator and the 3D columnar evaporator under one sun illumination.

	Bio-mimetic 3D evaporator	3D Columnar evaporator
Top Temperature (°C)	~ 33.6	~ 35.3
Bottom Temperature (°C)	~ 21.0	~ 20.2
Average Temperature (°C)	~ 28.9	~ 30.5
Water Evaporation in Darkness ($kg\ m^{-2}\ h^{-1}$)	0.84	0.62
Water Evaporation under One Sun Illumination ($kg\ m^{-2}\ h^{-1}$)	2.28	1.78
Energy Efficiency (%)	97.5	72.0

Supplementary Table 3

Supplementatry Table 3 | Material parameters for the numerical simulation process.

**Material properties**	**Symbol**	**Value**
heat conductivity coefficient of water	k_1	0.59 W m^{-1} K^{-1}
heat conductivity coefficient of air	k_2	0.026 W m^{-1} K^{-1}
heat conductivity coefficient of the 3D evaporator	k_3	0.19 W m^{-1} K^{-1}
diffusion coefficient of vapor	D	2.6×10^{-5} m^2 s^{-1}
saturated vapor concentration*	c_{sat}	0.8-1.6 mol m^{-3}
latent heat coefficient	L_v	2.4×10^6 J Kg^{-1}

*The saturated vapor concentration is temperature-dependent, the plot of saturated vapor concentration with temperature is shown in Supplementary Figure 20.

Supplementary Note 1

3D Printing Equipment: The 3D printing equipment is self-made with the LED UV projector (PRO4500, Wintech, China), liquid resin vat (self-made), supporting plate (self-made) mounting on a programmable moving platform (MC600, Zolix Instruments Co. Ltd., China) from bottom to up as displayed in Figure 1d. The UV projector can provide light patterns with a projection area of 51.6 mm × 32.2 mm, resolution of 912 × 1140 pixels, and light intensity range 0 - 65 mW cm^{-2}. The applicable velocity range of the programmable moving platform is 1.5 - 100 mm/min.

Supplementary Note 2

Characterization*:* Scanning electron microscope (SEM) images were obtained using a field-emission scanning electron microscope (SEM, JSM-7500F, JEOL, Japan). The surface oxygen content of the 3D printed evaporator was achieved through X-ray photoelectron spectroscopy (ESCALAB250XI, ThermoFisher Scientific, USA). Internal structure characterization was shot and reconstructed through a microcomputed tomography (Micro-CT) equipment (Skyscan 1272, Bruker, Germany). The resolution of single pixel is 3.0 μm. For the measurement of contact mode of water with the structure, the structure is loaded in a watch glass containing a thin layer of liquid, with the watch glass mounted on the sample stage. To reduce the influence of the liquid evaporation, ethylene glycol was employed as the test liquid due to its high boiling point. Individual X-ray exposure slices constructed the 3D copy of the sample. Rotation of the color-coded 3D model by the software allows a detailed morphology of the solid-water-air interface to be seen. Real time infrared thermal images were acquired through using an infrared camera (FLIR A655sc, USA) to investigate the temperature distribution and its evolution on the water/structure interface. The temperature profiles of different interfaces were measured under one sun illumination for 30 min. The experiments were typically conducted at an ambient temperature of 25.0 °C and humidity of ~ 50%. Simulated sunlight with an intensity of 1 kW m^{-2} (one sun, xenon arc lamp) was used, which was measured by a dynamometer (PM100D, Thorlabs, USA). For 3D evaporators with different H/D ratios, the top projected plane is utilized to normalize the light intensity. The water mass change was monitored by an electrical balance every minute. The projected area is used for the calculation of the mass change per area. The concentration of metal ions in the liquid was characterized by inductively coupled plasma mass spectroscopy (ICP-MS, Thermo iCAP RQ, USA) diluted by nitric acid.

Supplementary References

1. Zhao, F. *et al.* Highly efficient solar vapour generation via hierarchically nanostructured gels. *Nat. Nanotechnol.* **13**, 489-495 (2018).
2. Li, X. *et al.* Enhancement of Interfacial Solar Vapor Generation by Environmental Energy. *Joule* **2**, 1331-1338 (2018).
3. Shi, L. *et al.* Multi-functional 3D honeycomb ceramic plate for clean water production by heterogeneous photo-Fenton reaction and solar-driven water evaporation. *Nano Energy* **60**, 222-230 (2019).
4. Cui, L. F. *et al.* High Rate Production of Clean Water Based on the Combined Photo-Electro-Thermal Effect of Graphene Architecture. *Adv. Mater.* **30**, 1706805 (2018).
5. Xu, N. *et al.* A water lily–inspired hierarchical design for stable and efficient solar evaporation of high-salinity brine. *Sci. Adv.* **5**, eaaw7013 (2019).
6. He, S. M. *et al.* Nature-inspired salt resistant bimodal porous solar evaporator for efficient and stable water desalination. *Energy Environ. Sci.* **12**, 1558-1567 (2019).
7. Finnerty, C., Zhang, L., Sedlak, D. L., Nelson, K. L. & Mi, B. X. Synthetic Graphene Oxide Leaf for Solar Desalination with Zero Liquid Discharge. *Environ. Sci. Technol.* **51**, 11701-11709 (2017).
8. Kuang, Y. *et al.* A High‐Performance Self‐Regenerating Solar Evaporator for Continuous Water Desalination. *Adv. Mater.* **31**, 1900498 (2019).
9. Shi, Y. *et al.* Solar Evaporator with Controlled Salt Precipitation for Zero Liquid Discharge Desalination. *Environ. Sci. Technol.* **52**, 11822-11830 (2018).
10. Xia, Y. *et al.* Spatially isolating salt crystallisation from water evaporation for continuous solar

steam generation and salt harvesting. *Energy Environ. Sci.* **12**, 1840-1847 (2019).

11. Xu, W. C. *et al.* Flexible and Salt Resistant Janus Absorbers by Electrospinning for Stable and Efficient Solar Desalination. *Adv. Energy Mater.* **8**, 1702884 (2018).
12. Li, W., Li, Z., Bertelsmann, K. & Fan, D. E. Portable Low-Pressure Solar Steaming-Collection Unisystem with Polypyrrole Origamis. *Adv. Mater.* **31**, 1900720 (2019).
13. Shi, Y. S. *et al.* A 3D Photothermal Structure toward Improved Energy Efficiency in Solar Steam Generation. *Joule* **2**, 1171-1186 (2018).
14. Ni, G. *et al.* A salt-rejecting floating solar still for low-cost desalination. *Energy Environ. Sci.* **11**, 1510-1519 (2018).
15. Zhou, X. Y., Zhao, F., Guo, Y. H., Zhang, Y. & Yu, G. H. A hydrogel-based antifouling solar evaporator for highly efficient water desalination. *Energy Environ. Sci.* **11**, 1985-1992 (2018).
16. Liu, Z. *et al.* Continuously Producing Watersteam and Concentrated Brine from Seawater by Hanging Photothermal Fabrics under Sunlight. *Adv. Funct. Mater.* **29**, 1905485 (2019).
17. Vesel, A., Mozetic, M. & Zalar, A. XPS study of oxygen plasma activated PET. *Vacuum* **82**, 248-251 (2007).
