# Supplementary material for: Highly efficient three-dimensional solar evaporator for high salinity desalination by localized crystallization
Source: Nat Commun. 2020 Jan 27;11:521. doi: 10.1038/s41467-020-14366-1 (PMC6985111; doi:10.1038/s41467-020-14366-1)
Supplement: Supplementary file 3 — Description of Additional Supplementary File [file 41467_2020_14366_MOESM3_ESM.docx]

File Name: Supplementary Movie 1

Description: Real-time monitoring of water ultra-fast spreading process on the bio-mimetic 3D evaporator.

File Name: Supplementary Movie 2

Description: Real-time monitoring of the temperature distribution on the bio-mimetic 3D evaporator during the whole solar steam generation process.

File Name: Supplementary Movie 3

Description: Real-time monitoring of the crystallized salt moving along with the supplemented water film upward to the apex position.

File Name: Supplementary Movie 4

Description: The removal of the localized crystal from the bio-mimetic 3D evaporator through leaning the structure.

File Name: Supplementary Movie 5

Description: Micro-CT characterization of the inner morphology of the detached free-standing salt from the apex position.

File Name: Supplementary Movie 6

Description: Real-time monitoring of the batch purification process of natural seawater samples continuously for nine hours.
